# Bridging the Gap: Access to Care, Diagnostic Concordance, and Self-Rated Health in Older Adults

**DOI:** 10.1093/geroni/igaf122.083

**Published:** 2025-12-31

**Authors:** Amélie Quesnel-Vallée, Divine-Favour Ofili, Isabelle Dufour

**Affiliations:** McGill University, Montreal, Quebec, Canada; McGill University, Montreal, Quebec, Canada; Sherbrooke University, Montreal, Quebec, Canada

## Abstract

Self-rated health (SRH) is a widely utilized indicator of population health, shaped by multiple domains. Prior research has established that functioning, diseases, and pain are the most influential contributors to SRH in Canada, particularly among older adults. However, the role of access to care in shaping the diagnosis domain—and by extension, its influence on SRH—remains underexplored. We use the TorSaDE cohort, a linked dataset combining five cycles of the Canadian Community Health Survey (CCHS 2007-2016) with administrative health data covering the entire population of the province of Québec, Canada, enabling comprehensive analysis of health trajectories and outcomes among community-dwelling older adults. Using these linked data, we categorize participants into three diagnostic groups: (1) self-reported diagnosis only, (2) administrative (algorithmic) diagnosis only, and (3) concordant diagnosis across both sources. We conducted these analyses for both diabetes and major neurocognitive disorders (MNCD), and we examined the differences in self-ratings of health between these groups, and across a range of sociodemographic characteristics. We found that individuals with only algorithmically detected diagnoses reported significantly better self-rated health compared to the other two groups. This pattern was consistent for both diabetes and MNCD. These findings provide strong support for the cognitive model of self-rated health, suggesting that individuals primarily base their health assessments on known, consciously acknowledged diagnoses. Furthermore, this reliance on known information appears to override other “objective” health indicators, such as the intensity of health care utilization (as algorithmic detection inherently requires significant interaction with the health care system).

